# Seasonal and Antioxidant Evaluation of Essential Oil from *Eugenia uniflora* L., Curzerene-Rich, Thermally Produced in Situ

**DOI:** 10.3390/biom10020328

**Published:** 2020-02-19

**Authors:** Jamile S. da Costa, Adenilson S. Barroso, Rosa Helena V. Mourão, Joyce Kelly R. da Silva, José Guilherme S. Maia, Pablo Luis B. Figueiredo

**Affiliations:** 1 Programa de Pós-Graduação em Ciências Farmacêuticas, Universidade Federal do Pará, Belém 66075-900, Brazil; jamile.s.costa@hotmail.com (J.S.d.C.); gmaia@ufpa.br (J.G.S.M.); 2 Laboratório de Bioprospecção e Biologia Experimental, Universidade Federal do Oeste do Pará, Santarém 68035-110, Brazil; adenilson.barroso@yahoo.com.br (A.S.B.); mouraorhv@yahoo.com.br (R.H.V.M.); 3 Programa de Pós-Graduação em Biotecnologia, Universidade Federal do Pará, Belém 66075-900, Brazil; joycekellys@ufpa.br; 4 Programa de Pós-Graduação em Química, Universidade Federal do Maranhão, São Luís 64080-040, Brazil; 5 Departamento de Ciências Naturais, Universidade do Estado do Pará, Belém 66050-540, Brazil

**Keywords:** myrtaceae, sesquiterpenes, volatile composition, curzerene, seasonal variation, DPPH, 2,2-diphenyl-1-picrylhydrazyl

## Abstract

The essential oil of *Eugenia uniflora* has been attributed anti-depressive, antinociceptive, antileishmanial, larvicidal, antioxidant, antibacterial, and antifungal activities. It is known that the cultivation of this plant can be affected by seasonality, promoting alteration in the oil composition and its biological activities. This study aims to perform the annual evaluation of the curzerene-type oil of *E. uniflora* and determine its antioxidant activity. The oil yield from the dry season (1.4 ± 0.6%) did not differ statistically from that of the rainy season (1.8 ± 0.8%). Curzerene, an oxygenated sesquiterpene, was the principal constituent, and its percentage showed no significant difference between the two periods: dry (42.7% ± 6.1) and rainy (40.8 ± 5.9%). Principal component and hierarchical cluster analyses presented a high level of similarity between the monthly samples of the oils. Also, in the annual study, the yield and composition of the oils did not present a significant correlation with the climatic variables. The antioxidant activity of the oils showed inhibition of DPPH radicals with an average value of 55.0 ± 6.6%. The high curzerene content in the monthly oils of *E. uniflora* suggests their potential for use as a future phytotherapeutic alternative.

## 1. Introduction

*Eugenia* L. is included among the other 130 genera of Myrtaceae, and comprises about 1000 species distributed mainly in Central and South America and the African continent [1,2]. In the phytogeographical domains of Brazil, 260 species of *Eugenia* are described in the Atlantic Forest, 89 in the Amazon Forest, and 86 in the Brazilian Cerrado (Savanna) area [3], i.e., the country’s three main biomes. Among the taxa of Myrtaceae, *Eugenia* stands out for the diversity of its biological activities, for example, fungicide [4], bactericide [5], antioxidant, and anti-acetylcholinesterase [6].

*Eugenia uniflora* L. is a fruit tree which occurs naturally throughout South America, with widespread use of its fruits [7,8]. It is popularly known as “Pitanga”, a denomination originating in the Brazilian indigenous Tupi language, which means “dark red fruit”, and is also called “Cereja Brasileira” or “Ginja” [9]. The plant is used by traditional communities to treat diarrhea, cough, and rheumatism, and has presented other biological properties, such as hypothermic, antinociceptive, and antimicrobial activities [10,11]. Fruits of Pitanga are rich in calcium, magnesium, phosphorus, potassium, and vitamin C [12], as well as anthocyanins, flavonols, and carotenoids [13]. The fruits are consumed in their natural form, or in soft drinks, juices, ice-creams, sweets, liqueurs, and jellies, due to their sweet taste [14]. Traditional communities use an infusion of the leaves and barks as a antihypertensive and antipyretic, as well as against influenza, cough, cardiovascular diseases, asthma, sinusitis, colic, diarrhea, dysentery, fever, and rheumatism [10,11].

*Eugenia uniflora* is one of the most investigated species of the *Eugenia* genus, including through botanical, phytochemical, and pharmacological studies, due to its wide geographic distribution [15]. In a previously published paper, we reported that the essential oil of a curzerene-rich *E. uniflora* chemotype showed a significant antiproliferative and cytotoxic effect against some cancer strains [16]. Curzerene is a sesquiterpene hydrocarbon that was originally isolated from *Curcuma longa* L., a traditional Chinese herbal medicine, which showed anticancer activity in in vitro and in vivo models [17]. Also, curzerene was reported in the literature as a constituent produced by heating during the essential oil extraction and throughout the gas chromatographic analysis, resulting from the sigmatropic rearrangement of furanodiene, a heat-sensitive sesquiterpene compound which is present in the *E. uniflora* oil [18,19].

The objective of the present work was to evaluate the annual variability of the oil composition of *E. uniflora* leaves, the chemotype rich in curzerene thermally produced in situ, as a contribution to its possible use as an anticancer agent. To monitor the seasonal variability of the plant, tests were also carried out on the antioxidant activity of the oils produced, in addition to the previously observed cytotoxic effects.

## 2. Materials and Methods

### 2.1. Plant Material and Climatic Data

The leaves of *E. uniflora* were collected from a specimen existing on the Caratateua Island, Belém city, Pará state, Brazil (coordinates: 01°15′04.1” S/48°27′23.7” W). For the seasonal study, the leaves were sampled on day 5 of each month, at 8 am, from October 2017 to September 2018. Plant identification was performed by comparison with an authentic specimen of *Eugenia uniflora*, and a plant sample (MG 228493) was incorporated into the Herbarium “João Murça Pires” of Museu Paraense Emílio Goeldi, Belém city, State of Pará, Brazil. At the same time, the climatic parameters (radiation, relative air humidity, and rainfall precipitation) of the mentioned area were obtained each month from the website of the Instituto Nacional de Meteorologia (INMET, http://www.inmet.gov.br/portal/), of the Brazilian Government. Meteorological data were recorded through the automatic station A-201, at the Belém city, Pará state, Brazil, which is equipped with a Vaisala system, model MAWS 301 (Vaisala Corporation, Helsinki, Finland).

### 2.2. Essential Oils Extraction and Composition

The air-dried leaves were ground and subjected to hydrodistillation using a Clevenger-type apparatus (3 h). The oils were dried over anhydrous sodium sulfate, and the dry plant weights were used to calculate their yields. The moisture content of the plant samples was calculated using an infrared moisture balance for water loss measurement. Analysis of oil yield was done in triplicate.

The essential oil was dissolved with n-hexane at a concentration of 1500 µg/mL (3: 500, *v*/*v*), and analyzed simultaneously using gas chromatography–flame ionization detector (GC-FID, Shimadzu Corporation, Tokyo, Japan) and gas chromatography–mass spectrometry (GC/MS, Shimadzu Corporation, Tokyo, Japan) systems. The analysis of the oils was performed on a GCMS-QP2010 Ultra system (Shimadzu Corporation, Tokyo, Japan), equipped with an AOC-20i auto-injector and the GCMS-Solution software containing the NIST and FFNSC 2 libraries [20,21]. A Rxi-5ms (30 m × 0.25 mm; 0.25 μm film thickness) silica capillary column (Restek Corporation, Bellefonte, PA, USA) was used. The conditions of analysis were as follows. Injector temperature: 250 °C; Oven temperature programming: 60–240 °C (3 °C/min); Helium as carrier gas, adjusted to a linear velocity of 36.5 cm/s (1.0 mL/min); split mode injection for 1.0 μL of essential oil solution (6 µg of essential oil injected); split ratio 1:20 (300 ng to column for analysis); ionization by electronic impact at 70 eV; ionization source and transfer line temperatures of 200 and 250 °C, respectively. The mass spectra were obtained by automatic scanning every 0.3 s, with mass fragments in the range of 35–400 *m*/*z*. The retention index was calculated for all volatile components using a homologous series of C8-C40 n-alkanes (Sigma-Aldrich, Milwaukee, WI, USA), according to the linear equation of Van Den Dool and Kratz [22]. Individual components were identified by comparing their retention indices and mass spectra (molecular mass and fragmentation pattern) with those existing in the GCMS-Solution system libraries [20,21,23]. The quantitative data regarding the volatile constituents were obtained using a GC 2010 Series, operated under similar conditions to those of the GC-MS system. The relative amounts of individual components were calculated by peak-area normalization using a flame ionization detector (GC-FID) [16]. GC-FID and GC-MS analyses were performed in duplicate.

### 2.3. Antioxidant Assay

The antioxidant activity of the oils of seasonal samples was evaluated by the DPPH radical-scavenging method [24,25]. DPPH is a stable dark violet free radical with maximum absorption at 517 nm (Shimadzu, UV 1800, Shimadzu Corporation, Tokyo, Japan), which is reduced in the presence of antioxidants. A stock solution of 2,2-diphenyl-1-picrylhydrazyl (DPPH; 0.5 mM) was prepared in ethanol. The solution was diluted to approximately 60 µM, measuring an initial absorbance of 0.62 ± 0.02, at 517 nm and room temperature. The absorbance was measured at the start of the reaction, every 5 min during the first 30 min, and then at 30 min intervals until constant absorbance values were observed (plateau of reaction, 2 h). Each essential oil sample from the seasonal study (5.0 µL, 10 mg/mL) was mixed with Tris-HCl buffer (100 mM, 900 µL, pH 7.4), ethanol (40 µL), and Tween 20 solution (0.5%, 50 µL, *w*/*w*), and then added to DPPH (0.5 mM, 1 mL) in ethanol. The standard curves were prepared using Trolox (6-hydroxy-2,5,7,8-tetramethylchroman-2-carboxylic acid) (Sigma-Aldrich, St. Louis, MO, USA), at concentrations 30, 60, 150, 200, and 250 µg/mL. The results were expressed as milligrams of Trolox (mg TE/g) equivalents per gram of the sample.

### 2.4. Statistical Analysis

Statistical significance was assessed by a Tukey test (*p* < 0.05), and the Pearson correlation coefficients (R) were calculated to determine the relationship among the parameters analyzed (solar radiation, relative air humidity, and rainfall precipitation) in GraphPad Prism, version 5.0. A principal component analysis (PCA) was applied to verify the interrelation in the composition of the oils of the leaves (Minitab free 390 version, Minitab Inc., State College, PA, USA). A hierarchical grouping analysis (HCA), considering the Euclidean distance and complete linkage, was used to verify the similarity of the oil samples, based on the distribution of the constituents selected in the PCA analysis.

## 3. Results and Discussion

### 3.1. Essential Oil Yield vs Environmental Conditions

**Figure 1:**
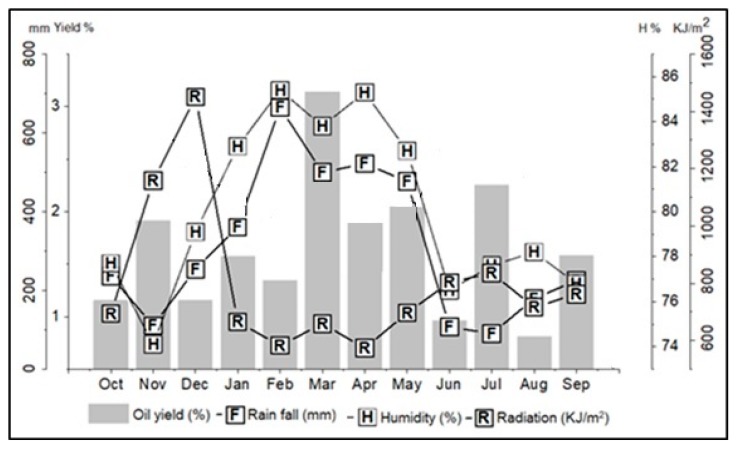
Relationship between the climatic parameters and the oil yield of *Eugenia uniflora* during the seasonal study.

Climatic parameters such as solar radiation, precipitation, and relative humidity were monitored for a period of one year (October 2017 to September 2018) to evaluate the influence of seasonality on yield and composition of *E. uniflora* essential oil. Mean values of solar radiation ranged from 573.4 KJ/m^2^ (April) to 1450.7 KJ/m^2^ (December), relative humidity from 74.1% (November) to 85.4% (February), and the average rainfall from 90.0 mm (July) to 664.4 mm (February). Based on the precipitation data, the rainy season occurred from December (360.8 mm) to April (664.4 mm), and the dry season from June (236.6 mm) to November (90.0 mm). May (476.6 mm) was a period of transition between these two seasons (Figure 1).

The Brazilian Amazonian climate is characterized only by a dry and a rainy season. Due to the permanent humid and warm climate, the Amazon presents spatial and seasonal heterogeneity of rainfall. The city of Belém, the collection site of *E. uniflora*, located in the Amazon region of northern Brazil, presents a higher rainfall index in December to April (rainy season) and a lower one in June to November (dry period). However, from one year to another, these two seasons may change, depending on the atmospheric phenomena that affect tropical regions [26].

In the annual study, the oil yield of *E. uniflora* ranged from 0.8% (August) to 3.1% (March), with an average of 1.7 ± 0.6% for the study period (see Table 1). Statistically (Tukey test), there was no significant difference in oil yields in the dry (1.4 ± 0.6%) and rainy (1.8 ± 0.8%) seasons. Additionally, significant correlation (*p* > 0.05) was not observed between the essential oil content and the climatic parameters, i.e., precipitation (R = 0.31), solar radiation (R = −0.17), and relative humidity (R = 0.32). Therefore, the results indicate that the variation in oil yields of *E. uniflora*, sampled in Caratateua Island, City of Belém, State of Pará, Brazil, do not correlate with climatic conditions and seasonal period.

### 3.2. Oil Composition and Environmental Conditions

The constituents of the oils were identified and quantified by GC-MS and GC-FID, respectively. Sixty-one compounds were identified, representing an average of 78.1% of the composition of the total oils (Table 1). Oxygenated sesquiterpenes were predominant (49.6–78.8%), followed by sesquiterpenes hydrocarbons (6.4–27.1%) and monoterpene hydrocarbons (0.5–8.6%).

**Figure 2:**
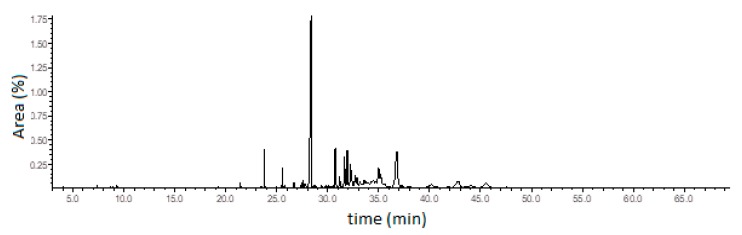
Ion-chromatogram of the *E. uniflora* oil.

**Figure 3:**
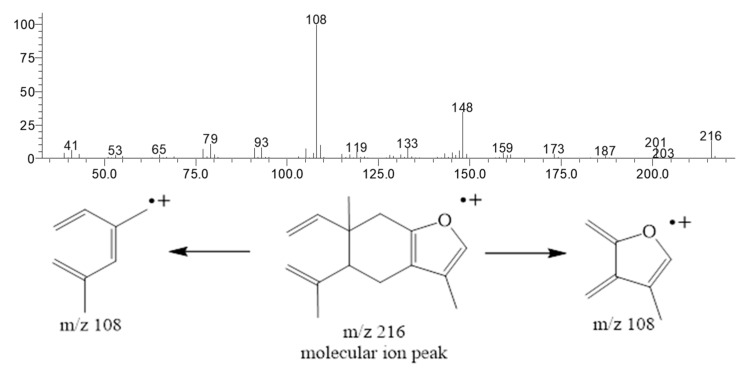
Curzerene mass spectrum and its base-peak fragmentation (108 *m*/*z*).

Curzerene, a germacrane-type oxygenated sesquiterpene, was the principal constituent of this *E. uniflora* oil during the study period. The percentage of curzerene varied from 34.4% (April) to 53.1% (August). The ion chromatogram of the *E. uniflora* oil and the curzerene mass spectrum showing the base-peak (108 *m*/*z*) fragmentation are depicted in Figure 2 and Figure 3 and the mass spectra and retention index of the main compound are showed in Table S1 (see Supplementary Materials).

The average content of curzerene during the dry (42.7% ± 6.1) and rainy (40.8 ± 5.9%) seasons did not present a statistically significant difference (Tukey test, *p* > 0.05). In addition, the climatic variables relative humidity (R = −0.07), solar radiation (R = −0.19), and precipitation (R = −0.03) showed no significant correlation with curzerene content (*p* > 0.05).

**Figure 4:**
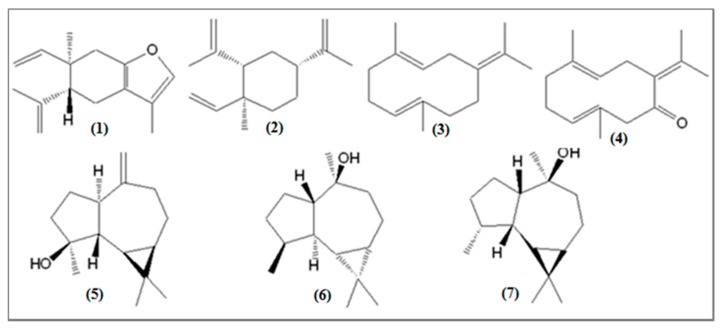
Other sesquiterpene compounds identified in the oils of *Eugenia uniflora*: (**1**) curzerene, (**2**) β-elemene, (**3**) germacrene B, (**4**) germacrone, (**5**) spathulenol, (**6**) globulol, (**7**) viridiflorol.

Other constituents were also identified in significant quantities in the *E. uniflora* oil (see Figure 4), such as the oxygenated sesquiterpenes germacrone (0.2–10.5%), globulol (1.5–7.4%), spathulenol (0.5–7.0%), and viridiflorol (0.8–6.2%), and the sesquiterpene hydrocarbons germacrene B (0.1–7.5%) and β-elemene (1.8–5.8%).

Based on the Pearson (R) correlation coefficient analysis, there was no statistically significant correlation (*p* > 0.05) between the main constituents of the oils and the climatic parameters (see Table 2). The results suggest that the variation in the yield and composition of the oils is not related to climatic factors.

**Figure 5:**
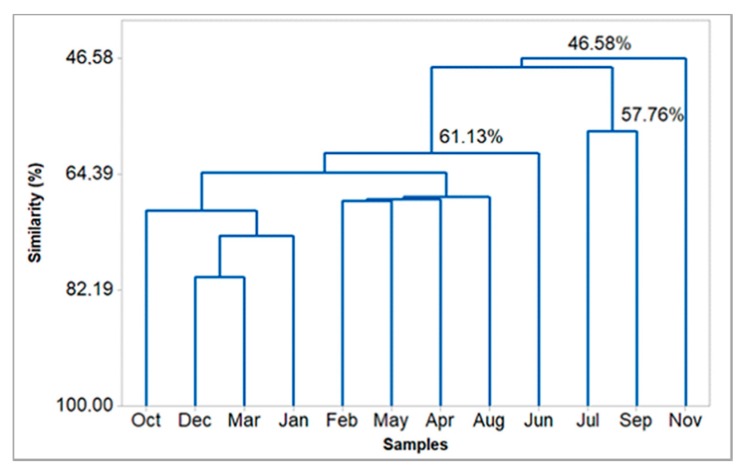
Hierarchical clustering analysis (HCA) of the *Eugenia uniflora* oils, based on their main constituents.

The application of a hierarchical clustering analysis (HCA) provided the dendrogram presented in Figure 5, which shows that the composition of the analyzed oils presented a similarity of 46.58%. HCA and PCA were plotted, with the constituents of oils displaying values above 3%.

**Figure 6:**
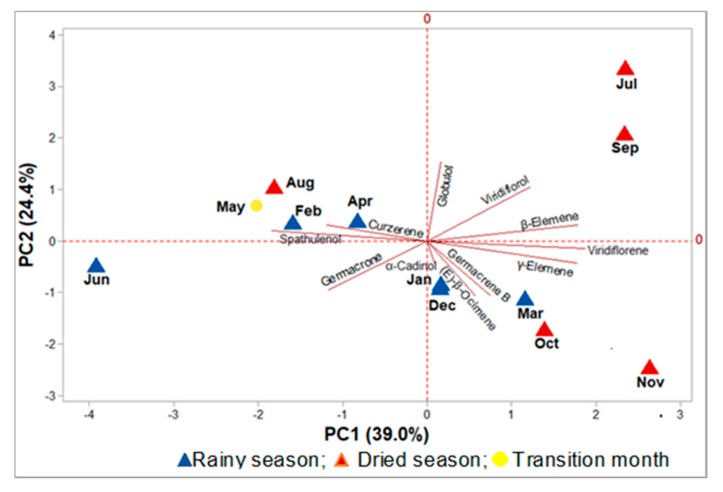
Principal components analysis (PCA) of the *Eugenia uniflora* oils, based on their primary constituents.

In the principal component analysis (PCA), the main components (PC1 and PC2) accounted for 63.4% of the total data variability, of which PC1 explained 39.0% and displayed positive correlations with the variables globulol (R = 0.04), viridiflorol (R = 0.28), β-elemene (R = 0.42), viridiflorene (R = 0.44), γ-elemene (R = 0.41), germacrene B (R = 0.18), (*E*)-β-ocimene (R = 0.13), and α-cadinol (R = 0.02). The component PC2 explained 24.4% of the variability, and showed a positive correlation with the compounds spathulenol (R = 0.08), curzerene (R = 0.11), globulol (R = 0.57), viridiflorol (R = 0.39), and β-elemene (R = 0.11). The PCA showed that there was no separation between the oil samples from the dry and rainy periods (Figure 6).

It is known that in addition to environmental factors, differences in the yield and composition of the oils can be attributed to genetic and geographic factors, invading predators (herbivory and pathogens), as well as soils (edaphic factors) [27]. In the study of specimens of *Eugenia lutescens* and *E. langsdorffii* from the Brazilian Midwest, the differences observed in the yield and oil composition were attributed to genetic variation and environmental factors, considered to be responsible for the production and variability of the metabolites identified in these two plants [28]. The leaves of *Eugenia neonitida* and *E. rotundifolia* were collected in Rio de Janeiro, Brazil every quarter. The primary compounds of the *E. neonitida* oil were bicyclogermacrene, germacrene, and β-caryophyllene. In *E. rotundifolia* oil, the main components were α-pinene, β-pinene, and β-caryophyllene. Seasonality did not significantly interfere in the oils of either species, despite the observed qualitative and quantitative variations [29].

In previous studies, yields of 0.5% and 1.8% for *E. uniflora* oil were reported in two samples collected in north and south Brazil, respectively [30,31]. A seasonal study of the oil of a specimen of *E. uniflora* from the Brazilian Midwest indicated that its oil yield in the rainy period (0.3 ± 0.3%) was similar to that of the dry period (0.2 ± 0.2%). On the other hand, it also indicated the existence of two groups of constituents: the group I of samples collected in the dry season, characterized by the presence of spathulenol and caryophyllene oxide; and the group II composed of samples collected in the rainy season, with a predominance of selina-1,3,7(11) trien-8-one epoxide. This work presented different results due to the influence of seasonality in the composition and percentage of the oil constituents [32]. Some *Eugenia uniflora* oils from the Amazon region showed differences in their composition which were attributed to the environmental factors at the collection sites, leading to the identification of four different chemical types: (1) selin-1,3,7(11)-trien-8-one and selin-1,3,7(11)-trien-8-one epoxide; (2) selin-1,3,7(11)-trien-8-one, selin-1,3,7(11)-trien-8-one epoxide and caryophyllene oxide; (3) curzerene; and (4) germacrene B, curzerene and β-caryophyllene [16].

In the present work with *E. uniflora* oil, seasonality did not influence curzerene content during the two seasonal periods (dry and rainy) of the Brazilian Amazon, as mentioned (see Figure 5). Curzerene was reported in the literature as a furanediene transformation product, which, as one of the volatile constituents of *E. uniflora* leaves, undergoes a sigmatropic thermal rearrangement (3,3 Cope rearrangement) upon oil distillation or conventional gas chromatographic analysis [18,19]. In a previous work, furanodiene was isolated by silica column chromatography of young leaf oil from an *E. uniflora* specimen collected in Rio de Janeiro, Brazil. However, it was not detected after conventional gas chromatography analysis (GC-FID) after heating at the injector port, which means that its conversion to curzerene was complete during the chromatographic run [33]. Also, from the oil of an *E. uniflora* specimen from Nigeria, Africa, furanodiene and curzerene were identified in almost equal proportions [34]. Furanediene was not identified by ^13^C-NMR in the oil of the present *E. uniflora* specimen. On the other hand, using column chromatography, eluted with n-hexane, it was possible to isolate curzerene in high purity, which means that during the oil distillation process, there was a complete transformation of furanediene into curzerene.

### 3.3. Antioxidant Capacity

Different methods of determining the antioxidant capacity of essential oils and their constituents have been described; each one depends on a specific generator of free radicals and reactions by different mechanisms [35]. For this work, the DPPH radical scavenging method was selected.

The *E. uniflora* oils, resulting from twelve months of collection of plant samples, showed a DPPH radical scavenging capacity with an average of 55.0 ± 6.6%, as shown in Table 2. The reaction kinetics were considered slow, with an average of 120 min. The highest percentage of inhibition of the DPPH radical was observed for the oils samples resulting from the collection on December (64.0%) and January (64.2%). The total antioxidant capacity was expressed in values equivalent to the standard Trolox. The oil samples from December (435.0 ± 0.6 mg TE/g) and January (436.3 ± 2.3 mg TE/g) showed antioxidant activity only twice as low as Trolox, and therefore, with significant values.

The oils of *E. uniflora*, which were extracted in June, October, and November, inhibited the DPPH radical in a statistically similar manner (*p* > 0.05), presenting a similarity level of 46.58% (see Figure 5). Also, the oils obtained in December and January were statistically similar, as justified by the convergent composition observed in the HCA plot, with a similarity of 73.81%. The similarity of the December and January oils was confirmed in the PCA plot, where there is a superposition of both points (see Figure 6). The oils extracted in February and March also showed similar inhibition of the DPPH radical, exhibiting 64.05% similarity in their compositions, as observed in the HCA plot. In April and May, April and July, and April and September, the corresponding oils inhibited the DPPH radical, presenting similarity levels of 68.2%, 48.0%, and 48.0%, respectively. On the other hand, the oil obtained in August did not similarly inhibit the DPPH radical, showing the lowest inhibition (42.6%, 186.9 mg ET/g). In addition, the antioxidant activity showed a moderate and negative correlation with curzerene (R = −0.52) and the monoterpene hydrocarbons (−0.56) contents, as well as a weak or negligible correlation with climatic variables (R < 0.5).

A significant antioxidant activity of curzerene and germacrone against the DPPH radical was previously reported for some *Curcuma* oils, using the bioautography method [36]. The antioxidant activity of essential oils should not be considered only vis-a-vis its primary constituents. The synergistic action of other minor components contributes to an increase in antioxidant activity [37]. For instance, in two chemotypes of *E. uniflora* oils, this effect was observed in early reports [16]. One of them showed selin-1,3,7(11)-trien-8-one, selin-1,3,7(11)-trien-8-one epoxide, and caryophyllene oxide as significant constituents of the oil. The other chemotype presented a high curzerene content in the oil. The inhibition rates of the DPPH radical in oils were 45.1% and 42.8% for these two chemotypes, respectively [16]. Another oil of *E. uniflora*, mainly composed by germacrene B and selin-1,3,7(11)-trien-8-one epoxide, presented a significant DPPH antioxidant activity (IC_50_ 833.3 ± 20.7 μg/mL), based on the DPPH scavenging assay [36]. The antioxidant capacity of the oils of different species of *Eugenia* has been reported, e.g., *E. flavescens* oil, with a value of 122.6 ± 6.8 mg TE/mL, *E. patrisii* oil, with a value of 111.2 ± 12.4 mg TE/mL), and *E. egensis* oil, with a value of 216.5 ± 11.6 mg TE/mL) value [24].

## 4. Conclusions

The results showed that climatic variables did not influence the average yield and curzerene content of *E. uniflora* essential oil. Environmental factors also had little influence on the composition of the oil and its antioxidant potential. The high curzerene content in this *E. uniflora* sample suggests its potential as a renewable source of this biologically-active compound, in particular as an antioxidant and antitumor agent. The study of this specimen contributes to the knowledge of *E. uniflora*, which is already widely used for its medicinal potential. Considering that there was a qualitative variation in the composition of the essential oil, with the transformation of furanodiene into curzerene by thermal rearrangement, this situation should be considered in the standardization of the oil, given its use as a possible herbal product.

## Figures and Tables

**Table 1:** Oil-composition of seasonal study of *Eugenia uniflora*.

Oil Yields (%)			1.6	1.3	3.1	1.9	2.0	1.0	2.2	0.8	1.6	1.2	1.9	1.2
Oil Constituents (%) *	RI_C_	RI_L_	January	February	March	April	May	June	July	August	September	October	November	December
Hexan-3-one	785	782 ^a^	0.1				0.1							
Hexan-2-one	788	786 ^b^	0.1	0.1		0.1	0.1	0.1	0.1					
Hexan-3-ol	792	795 ^b^	0.1	0.1	0.1	0.1	0.1	0.1	0.1					
Hexan-2-ol	795	802 ^a^	0.1	0.1	0.1	0.1	0.1	0.2						
Hexen-2*E*-al	847	846 ^a^	0.1	0.1	0.1	0.1	0.1	0.3	0.1	0.1	0.1	0.1	0.2	0.1
Hexen-3*Z*-ol	849	850 ^a^		0.1	0.1					0.1		0.1	0.1	
Myrcene	991	988 ^a^	0.1	0.5	0.3	0.2	0.4	1.1	0.1	0.3	0.2	0.5	1.6	0.2
β-Phellandrene	1028	1025 ^a^	0.1		0.1							0.3	0.7	
Limonene	1028	1024 ^a^		0.3		0.2	0.2	0.5	0.1	0.2	0.2			0.1
(*Z*)-β-ocimene	1036	1032 ^a^	0.1	0.5	0.4	0.1	0.1	0.6	0.1	0.4	0.1	0.6	2.0	0.2
(*E*)-β-ocimene	1046	1044 ^a^	0.2	1.1	1.0	0.2	0.2	1.0	0.1	1.1	0.2	1.5	4.3	0.6
Linalool	1099	1095 ^a^		0.1				0.1	0.1		0.1	0.1	0.1	
Hexen-3*Z*-yl butanoate	1185	1184 ^a^		0.1		0.1			0.1		0.1	0.1	0.1	
δ-Elemene	1336	1335 ^a^	0.5	0.4	0.9	0.4	0.3	0.1	0.7	0.6	0.6	1.2	1.6	0.7
Isoledene	1374	1374 ^a^	0.1	0.1	0.1	0.1			0.1		0.1	0.1	0.1	
α-Copaene	1376	1374 ^a^							0.1		0.1	0.1	0.1	0.1
β-Elemene	1385	1389 ^a^	2.8	3.3	4.2	3.5	3.3	1.8	5.8	2.6	4.4	5.0	4.4	3.8
α-Gurjunene	1411	1409 ^a^	0.1	0.1	0.1	0.1			0.2		0.1	0.1	0.2	0.1
*E*-Caryophyllene	1420	1417 ^a^	0.1	0.1	0.2	0.1	0.1		0.3	0.1	0.2	0.3	0.3	0.3
β-Copaene	1428	1430 ^a^			0.1						0.1			0.1
γ-Elemene	1434	1434 ^a^	2.1	1.4	2.7	1.3	1.2	0.8	2.8	1.7	2.3	3.7	2.9	2.6
Aromadendrene	1440	1439 ^a^	0.4	0.2	0.4	0.3	0.2		0.6		0.4	0.4	0.4	0.3
Selina-5,11-diene	1444	1445 ^b^	0.1	0.1					0.1		0.1	0.1	0.1	0.1
Cadina-3,5-diene	1451	1452 ^b^			0.1							0.1		
*cis*-Muurola-3,5-diene	1452	1448 ^a^	0.1		0.1								0.1	
α-Humulene	1454	1452 ^a^				0.1			0.1			0.1	0.1	0.1
*allo*-Aromadendrene	1460	1458 ^a^				0.5		0.2		0.3				
9-*epi*-*E*-Caryophyllene	1462	1464 ^a^	0.5	0.4	0.7		0.5		1.0		0.8	0.7	0.8	0.5
Selina-4,11-diene	1476	1476 ^b^	0.4	0.2	0.4	0.2			0.5	0.2	0.4	0.4	0.5	0.3
Germacrene D	1482	1478 ^a^		0.8	2.5	0.6	0.4	0.5	0.9	1.5		2.7	2.8	1.7
γ-Muurolene	1481	1478 ^a^	1.4								1.0			
β-Selinene	1487	1481 ^a^	0.8	0.5	0.6	0.6	0.6		0.6	0.4	0.5	0.6	0.7	0.8
δ-Selinene	1492	1492 ^a^	0.2	0.1	0.3	0.1			0.2	0.1	0.1	0.3	0.4	0.2
Viridiflorene	1496	1496 ^a^	2.4	0.8	2.7	1.4	0.8	0.2	2.8	0.8	3.2	1.4	4.1	1.9
**Curzerene**	**1503**	**1499 ^a^**	**35.8**	**48.7**	**43.6**	**34.4**	**45.0**	**49.1**	**41.1**	**53.1**	**41.0**	**44.0**	**36.2**	**41.4**
α-Muurolene	1502	1500 ^a^		0.1	0.2	0.1	0.1			0.1		0.2		
Germacrene A	1504	1508 ^a^					0.1	0.2						
α-Bulnesene	1511	1509 ^a^		0.2					0.2		0.2	0.9	0.1	
γ-Cadinene	1516	1513 ^a^	0.1	0.1	0.1	0.1				0.3			0.1	0.1
7-*epi*-α-Selinene	1519	1520 ^a^										0.1	0.1	0.1
δ-Cadinene	1525	1522 ^a^	0.5	0.2	0.5	0.2	0.2	0.1	0.1	0.1	0.1	0.5	0.5	0.5
γ-Cuprenene	1536	1532 ^a^										0.1	0.1	
Selina-4(15),7(11)-diene	1538	1540 ^b^										0.3	0.3	0.1
Selina-3,7(11)-diene	1542	1546 ^b^								0.1	0.2	0.2		
Germacrene B	1559	1559 ^a^	4.8	3.9	6.6	3.5	3.5	2.8		0.1	5.9	7.5	5.0	6.0
Maaliol	1566	1566 ^a^					0.7							
Spathulenol	1578	1577 ^a^	1.7	4.2	0.8	5.4	7.0	6.9	1.2	3.3	1.6	1.7	0.5	3.4
Globulol	1583	1590 ^a^		2.7		4.1	3.6	1.5	7.4	2.1	5.1			
Viridiflorol	1592	1592 ^a^	2.7	1.5	1.9	2.4	2.0	1.0	4.7	0.8	6.2	1.3	1.8	1.9
Cubeban-11-ol	1594	1595 ^a^	1.1	0.5		0.7	0.6					0.5	0.7	0.6
Rosifoliol	1603	1600 ^a^	1.1	0.5	0.6	0.5	0.6	0.2				0.5	0.6	0.6
*trans*-β-Elemenone	1604	1602 ^a^	1.9	0.8	0.9	1.3	1.0	1.7		0.4		0.8	0.9	1.1
Junenol	1618	1618 ^a^										0.1	0.2	
γ-Eudesmol	1632	1630 ^a^										0.1	0.2	0.2
8-isobutyryloxy-Isoborneol	1638	1632 ^a^											0.4	
*epi*-α-Murrolol	1641	1640 ^a^										0.6	0.8	0.9
α-Muurolol	1646	1644 ^a^										0.1	0.3	0.3
α-Cadinol	1655	1652 ^a^				3.1	1.8					1.1	2.2	0.2
Atractylone	1657	1657 ^a^	1.1	1.4	0.9	1.3	1.2		1.3	0.1	0.5	0.7	0.8	0.8
Intermedeol	1666	1665 ^a^										0.1	0.2	
Germacrone	1696	1693 ^a^	7.5	4.2	4.8	5.6	5.2	10.5		0.2		4.2	3.8	5.4
Monoterpene hydrocarbons			0.5	2.4	1.8	0.7	0.9	3.2	0.4	2.0	0.7	2.9	8.6	1.1
Oxygenated monoterpenes				0.1				0.1	0.1		0.1	0.1	0.1	
Sesquiterpene hydrocarbons			17.4	13.0	23.5	13.2	11.3	6.4	17.1	9.0	20.8	27.1	25.8	20.4
Oxygenated sesquiterpenes			52.9	64.5	53.5	78.8	68.7	70.9	55.7	60.0	54.4	55.8	49.6	56.8
Others			0.5	0.6	0.4	0.5	0.5	0.7	0.4	0.2	0.2	0.3	0.4	0.1
Total			71.3	80.6	79.2	73.2	81.4	81.6	73.7	71.2	76.2	86.2	84.5	78.4

RI_C_ = Calculated Retention Index (Rxi-5ms column); RI_L_ = Literature Retention index; ^a^ = Adams; 2007 [22]; ^b^ = Mondello, 2011 [20]; Main constituents in bold; * The constituent amounts were calculated by peak-area normalization using a flame ionization detector (GC-FID).

**Table 2:** Antioxidant capacity of the monthly oils of *Eugenia uniflora*.

Months	Antioxidant Capacity	
	Trolox Equivalent (mg TE/g)	Inhibition (%) *
January	436.3 ± 2.3	64.2 ± 0.3 ^b^
February	378.2 ± 6.2	55.7 ± 0.9 ^c^
March	393.2 ± 4.8	57.9 ± 0.7 ^c^
April	371.1 ± 7.1	57.3 ± 0.4 ^c,d^
May	373.6 ± 13.1	55.0 ± 1.9 ^c,d,e^
June	339.4 ± 1.6	51.1 ± 3.1 ^a^
July	400.3 ± 3.4	59.6 ± 1.2 ^c,d,f^
August	186.9 ± 7.7	42.6 ± 0.3 ^h^
September	286.6 ± 4.0	57.9 ± 0.4 ^c,d,e,f,g^
October	322.3 ± 3.4	47.4 ± 0.5 ^a^
November	326.5 ± 10.3	48.1 ± 1.5 ^a^
December	435.0 ± 0.6	64.0 ± 0.1 ^b^

* Values are expressed as means ± standard deviations (n = 3). Values with the same letters in the column do not differ statistically in the Tukey test (*p* > 0.05).

## Supplementary Materials

The following are available online at https://www.mdpi.com/2218-273X/10/2/328/s1, Table S1: Mass spectra and retention index of the main compound identified in the essential oils of *Eugenia uniflora*.
