# Supplementary material for: Seasonal and Antioxidant Evaluation of Essential Oil from Eugenia uniflora L., Curzerene-Rich, Thermally Produced in Situ
Source: Biomolecules. 2020 Feb 19;10(2):328. doi: 10.3390/biom10020328 (PMC7072495; doi:10.3390/biom10020328)
Supplement: Supplementary file 1 [file biomolecules-10-00328-s001.pdf]

Table S1. Mass spectra and structures of main constituents identified in the oils of *Eugenia uniflora*.

Compound	Mass spectra
curzerene	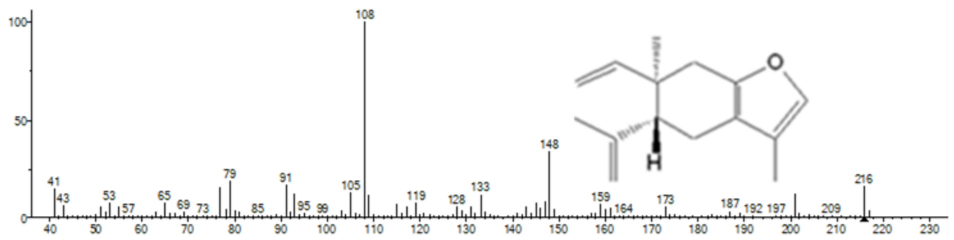
β-elemene	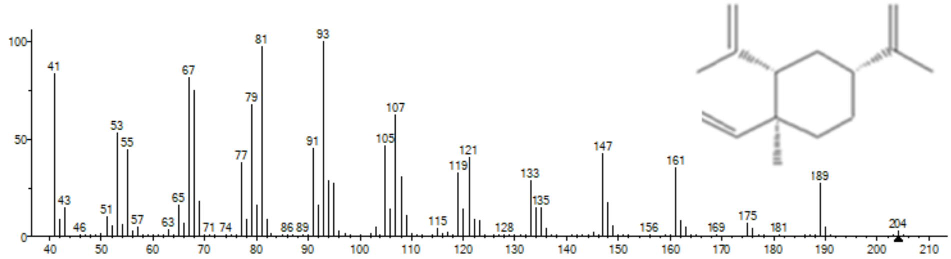
germacrene B	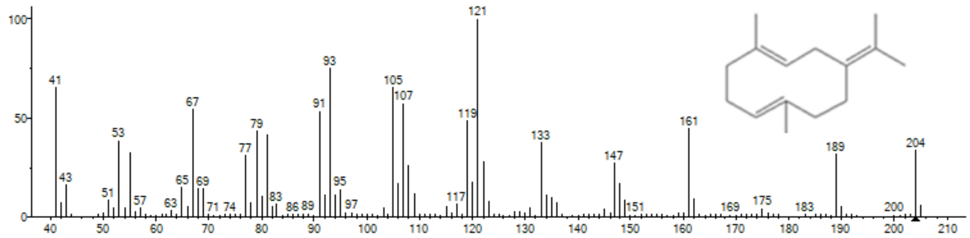
germacrone	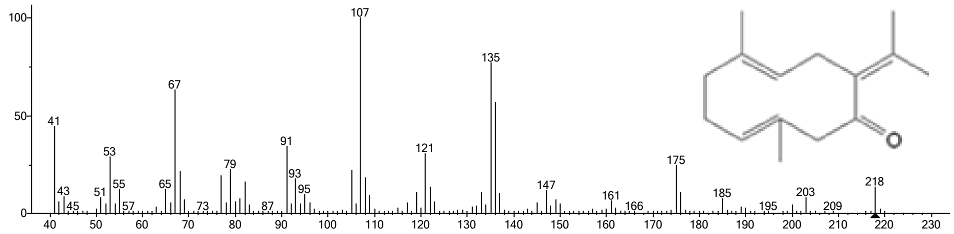
spathulenol	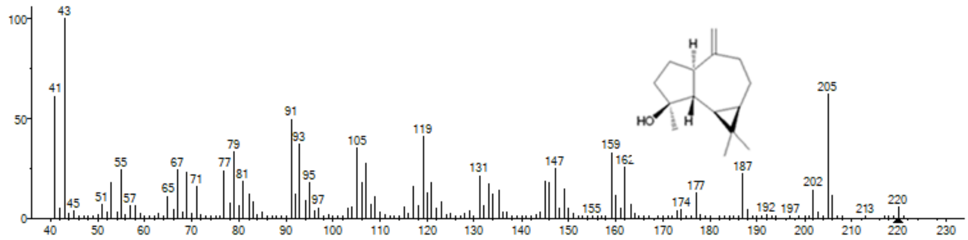
globulol	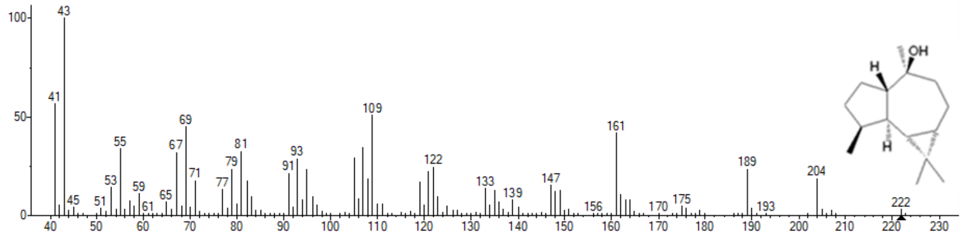

viridiflorol	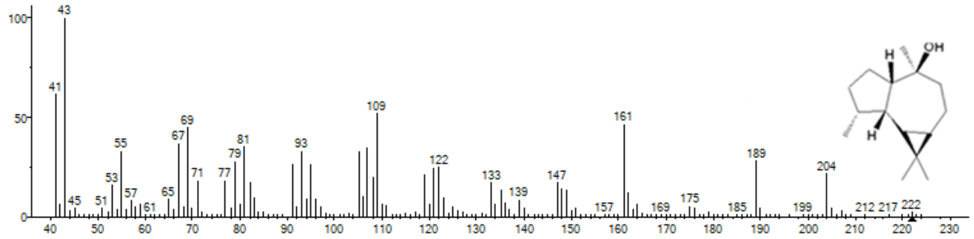
